# Revision Anterior Cruciate Ligament Reconstruction and Increased Tibial Slope: When to Perform a Slope-Altering High Tibial Osteotomy

**DOI:** 10.1177/26350254221102461

**Published:** 2022-09-06

**Authors:** Gian Andrea Lucidi, Bálint Zsidai, Philipp W. Winkler, Brian M. Godshaw, Jonathan D. Hughes, Volker Musahl

**Affiliations:** * Department of Orthopaedic Surgery, UPMC Freddie Fu Sports Medicine Center, University of Pittsburgh, Pittsburgh, Pennsylvania, USA; † Istituto Ortopedico Rizzoli, Istituto di Ricovero e Cura a Carattere Scientifico, Bologna, Italy; ‡ Department of Orthopaedics, Institute of Clinical Sciences, Sahlgrenska Academy, University of Gothenburg, Gothenburg, Sweden; § Department for Orthopaedic Sports Medicine, Klinikum rechts der Isar, Technical University of Munich, Munich, Germany; Investigation performed at Department of Orthopaedic Surgery, University of Pittsburgh Medical Center, Pittsburgh, Pennsylvania, USA

**Keywords:** revision anterior cruciate ligament reconstruction, high tibial osteotomy, graft failure, posterior tibial slope, ACL-R

## Abstract

**Background:**

Slope-correcting high tibial osteotomy (HTO) is gaining popularity for mitigating the impact of posterior tibial slope (PTS) on graft failure in patients requiring revision anterior cruciate ligament (ACL) reconstruction surgery. Biomechanical and clinical studies have demonstrated that PTS reduction results in decreased graft forces and satisfactory patient outcomes, making it an important technique in the setting of complex revision ACL reconstruction (ACL-R).

**Indications:**

Slope-correcting HTO can be performed for the management of recurrent knee instability after ACL-R due to a high PTS of 12° or greater.

**Technique Description:**

A 3- to 4-inch incision is made along the tibia using an anteromedial approach, and the exposed patellar tendon is protected using a retractor. The anterior compartment is exposed by an incision in the tibialis anterior fascia, followed by elevation of the tibialis anterior and placement of a Hohmann retractor. Subperiosteal elevation of the medial collateral ligament (MCL) is performed on the medial side of the tibial tubercle. The position of eventual screw fixation is marked, and a straight tubercle osteotomy is performed without anteriorization, leaving a freely exposed proximal tibia. Two K-wires are used to mark the location of the osteotomy. Soft tissue structures are protected with Hohmann retractors on both sides while using an oscillating saw to perform the osteotomy. A few degrees (1°-3°) of overcorrection are preferred. The osteotomy is completed, retaining 1cm of posterior hinge. The wedge is removed and reduced by hyperextending the knee under gentle manual traction. Pre-contoured, low-profile plates with locking screws are placed, with 3 screws proximally and another 3 distally. During concurrent ACL-R, the screws should leave room for drilling of the ACL tibial tunnel.

**Results:**

Studies investigating the effects of slope-correcting HTO concurrent to revision ACL-R have reported reduced anterior tibial translation and rotatory knee instability, improved patient-reported outcomes, and a reduction in graft failure risk.

**Description/Conclusion:**

Slope-reducing HTO is an essential technique in the arsenal of complex ACL surgeons aiming to correct the detrimental effect of an increased PTS on ACL graft integrity in the setting of revision ACL-R.

## Graphical abstract

**Graphical Abstract:**
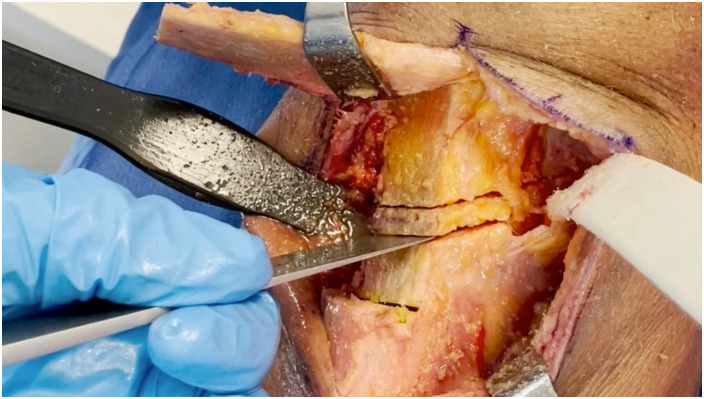
This is a visual representation of the abstract.

## Video Transcript

Hi! My name is Volker Musahl. I’m a Professor at the University of Pittsburgh and I’m presenting “Revision Anterior Cruciate Ligament Reconstruction and Increased Tibial Slope: When to Perform a Slope-Altering High Tibial Osteotomy.”

There are no disclosures relevant to this particular presentation.

Just a quick introduction: Different osteotomy options exist to provide solutions to different types of problems. These options include closing-wedge osteotomy, opening-wedge osteotomy for varus arthritis (which is probably the most commonly performed osteotomy currently), and deflexion osteotomy, which is the topic of the current presentation.

The general indications for deflexion osteotomy include an isolated management of osteoarthritis (OA) and malalignment. Slope correction can also be performed in the setting of instability. However, the expanded indications are more interesting. Examples include posterolateral corner reconstruction failure due to malalignment with double varus or malalignment with cartilage procedures and ACL-R failure due to an increased posterior tibial slope (PTS).^3^ The latter is especially interesting in the setting of a second revision.

Deflexion osteotomy can be performed according to 2 general methods. On the left, according to Sonnery-Cottet et al,^7^ an osteotomy through the tibial tubercle is shown. This method involves forward elevation of the tubercle, followed by repair. On the right, according to Dejour et al,^2^ osteotomy is performed above the tubercle, which does not require elevation of the tubercle.

Slope-reduction using anterior closing-wedge HTO is conducted for recurrent instability after ACL-R. The procedure can be considered for second or multiple revision cases, when a neutral leg-axis is present and the steepness of the tibial slope is approximately >12°.^4^ It is important to be aware of contraindications. Care must be taken with patients who have >10° of hyperextension in the knee, since osteotomy may contribute to an increase in hyperextension. In addition, deflexion osteotomy should not be performed when a varus deformity >5° is present. Varus malalignment needs to be addressed at the same time and is more appropriate to correct by a medial opening-wedge or a lateral closing-wedge osteotomy. Moreover, grade 4 osteoarthritis is also considered a general contraindication.

During preoperative planning, a detailed clinical examination and imaging are essential. Magnetic resonance imaging and other advanced imaging methods assessing concomitant pathology, such as root tears and meniscus or collateral ligament injury, should be performed. Specific to the osteotomy, an appropriate lateral radiograph should be obtained, with a perfect overlap of the femoral condyles. The PTS is measured using the circle method, which helps avoid overcorrection or undercorrection. Patient-specific instrumentation (PSI) is available from several sources and is helpful in conducting accurate and appropriate preoperative planning.

The following slide demonstrates some of the surgical instruments necessary for performing a slope-correcting osteotomy. An oscillating saw and multiple osteotomes are used, which can be seen on the lower left. Lamina spreaders are preferred to open the osteotomy gaps. Screwdrivers and a plate set are also required for subsequent fixation.

The patient is placed in a supine position, with an L-bar and side-post used for stability. With the knee in approximately 90° of flexion, a tourniquet is placed but not necessarily used. Examination under anesthesia is performed at the start of the case, followed by diagnostic arthroscopy. A large, sterilely draped C-arm is used to provide guidance during the case.

The procedure is generally performed using an anteromedial approach. A 3- to 4-inch incision is sufficient. The patellar tendon is exposed, followed by exposure of the medial collateral ligament (MCL). The patellar tendon can be observed, and the retractor is placed for protecting the tendon. Next, the anterior compartment is carefully exposed by an incision in the fascia of the tibialis anterior. The tibialis anterior is then elevated, and a Hohmann retractor is placed. This is followed by elevation of the MCL on the medial side of the tubercle. I prefer to perform a subperiosteal elevation of the MCL.

The position of eventual screw fixation of the tubercle is then marked, and a straight tubercle osteotomy is performed without anteriorization. The tubercle is then lifted off, leaving a free exposure to the proximal tibia. Two K-wires are used to mark the location of the planned osteotomy. An oscillating saw is used, and the tibia and soft tissue structures are protected with Hohmann retractors on both sides of the osteotomy. Radiolucent Hohmann retractors can be used. The size of the osteotomy somewhat depends on the end-goal, but in general, I prefer a few degrees of overcorrection. As an example, when aiming to achieve a 5° slope after starting with 15°, a 1-cm thick bone wedge is removed.

The osteotomy is then carefully completed using the osteotomes. One centimeter of posterior hinge is retained and is very important to leave intact. A second osteotome can then be inserted. Once both wedges are appropriately created, the osteotomy wedge may be removed and reduced. Reduction is achieved by simply hyperextending the leg. In the event of residual resistance, curettes, rongeurs, and sometimes a small drill-bit may be used for correction of the posterior hinge. The leg is then extended under gentle manual traction until the osteotomy gap is closed.

At this point, the only remaining step is the placement of hardware and screws. This process is not demonstrated in detail, but a pre-contoured, low-profile plate with locking screws is used, with 3 screws placed proximally and another 3 placed distally. It is important to note that when concurrently performing an ACL-R, the screws should be oriented in a way that leaves room for the ACL tibial tunnel to be drilled. This is possible to attain using the described approach. At the end of the case, the tibial tubercle is reduced in length corresponding to the thickness of the osteotomy wedge removed for slope reduction, to avoid patella baja. Fixation is performed using two 4.5-mm cortical screws in lag screw technique.

Among the potential complications of a slope-reducing HTO,^4^ wound infection and wound breakdown may occur, and may necessitate hardware removal. Care should be taken to avoid undercorrection or overcorrection of PTS, genu recurvatum, and coronal plane malalignment. Fluoroscopic guidance should be used to prevent hinge fracture of the posterior tibial cortex. Further serious complications include injury to the popliteal artery,^5^ compartment syndrome, and non-union of the osteotomy site.

General rehabilitation considerations for the first 4 weeks follow a conservative approach. Motion is restricted to 0° to 90° without weight-bearing. Following radiographic control, increased weight-bearing can be started with full weight-bearing at approximately 6 weeks. Light-impact aerobics are permitted at 12 weeks, followed by return to full sports activity or duty between 3 and 4 months after surgery.

The next slide shows outcomes based on data from Sonnery-Cottet et al,^7^ in Lyon. This a small series, published in the *American Journal of Sports Medicine* (AJSM). A slope correction from 14° to 9° was performed, resulting in good outcomes. Dejour et al^2^ published results in the *Knee Surgery, Sports Traumatology, Arthroscopy* journal at around the same time. This study included 9 patients who underwent slope correction of approximately 4°.

The next study from Germany includes 22 patients with an approximately 6° slope correction^1^ and reports reduction of anterior tibial translation, improved patient-reported outcome measures (PROMs), and no residual pivot shift in the study population. The final study was described by Song et al^6^ in AJSM and includes 18 patients with a 2- to 3-year follow-up. Approximately 10° of slope correction is reported with sufficient reduction of anterior tibial translation, no pivot shift, and no graft re-ruptures, in accordance with the previous studies.

The take-home message of this presentation is that slope-correcting osteotomy is a procedure to be considered when performing complex ACL revision surgeries. Assessment of the intrinsic risk factors of each patient is essential prior to any revision procedure. The tibial slope affects posterior tibial translation and rotatory stability in the ACL-deficient knee, which is important to take into account. The causes of ACL-R failure must always be investigated and are not limited to the characteristics of the tibial slope. Tunnel position and meniscus deficiency may also be contributing factors and should be addressed when needed. Slope-reducing HTO is a useful tool in the revision setting, so make it a part of your surgical repertoire.

Thank you very much for listening to this presentation.
